# TBLR1 regulates the expression of nuclear hormone receptor co-repressors

**DOI:** 10.1186/1471-2121-7-31

**Published:** 2006-08-07

**Authors:** Xin-Min Zhang, Qing Chang, Lin Zeng, Judy Gu, Stuart Brown, Ross S Basch

**Affiliations:** 1 Department of Pathology, New York University School of Medicine, New York, NY 10016, USA; 2 NYU Cancer Institute, New York University Medical Center, New York, NY 10016, USA; 3 Department of Cell Biology, New York University School of Medicine, New York, NY 10016, USA

## Abstract

**Background:**

Transcription is regulated by a complex interaction of activators and repressors. The effectors of repression are large multimeric complexes which contain both the repressor proteins that bind to transcription factors and a number of co-repressors that actually mediate transcriptional silencing either by inhibiting the basal transcription machinery or by recruiting chromatin-modifying enzymes.

**Results:**

TBLR1 [GenBank: NM024665] is a co-repressor of nuclear hormone transcription factors. A single highly conserved gene encodes a small family of protein molecules. Different isoforms are produced by differential exon utilization. Although the ORF of the predominant form contains only 1545 bp, the human gene occupies ~200 kb of genomic DNA on chromosome 3q and contains 16 exons. The genomic sequence overlaps with the putative DC42 [GenBank: NM030921] locus. The murine homologue is structurally similar and is also located on Chromosome 3. TBLR1 is closely related (79% homology at the mRNA level) to TBL1X and TBL1Y, which are located on Chromosomes X and Y. The expression of TBLR1 overlaps but is distinct from that of TBL1. An alternatively spliced form of TBLR1 has been demonstrated in human material and it too has an unique pattern of expression. TBLR1 and the homologous genes interact with proteins that regulate the nuclear hormone receptor family of transcription factors. In resting cells TBLR1 is primarily cytoplasmic but after perturbation the protein translocates to the nucleus. TBLR1 co-precipitates with SMRT, a co-repressor of nuclear hormone receptors, and co-precipitates in complexes immunoprecipitated by antiserum to HDAC3. Cells engineered to over express either TBLR1 or N- and C-terminal deletion variants, have elevated levels of endogenous N-CoR. Co-transfection of TBLR1 and SMRT results in increased expression of SMRT. This co-repressor undergoes ubiquitin-mediated degradation and we suggest that the stabilization of the co-repressors by TBLR1 occurs because of a novel mechanism that protects them from degradation. Transient over expression of TBLR1 produces growth arrest.

**Conclusion:**

TBLR1 is a multifunctional co-repressor of transcription. The structure of this family of molecules is highly conserved and closely related co-repressors have been found in all eukaryotic organisms. Regulation of co-repressor expression and the consequent alterations in transcriptional silencing play an important role in the regulation of differentiation.

## Background

TBLR1 is a transcriptional regulator interacting with the co-repressors of nuclear hormone receptor activity. We cloned the gene encoding this protein and identified it as a member of a small family of proteins that include at least two isoforms encoded by the same gene and closely related, X- and Y linked proteins, called TBL1X and TBL1Y. The TBLR1 protein interacts with NHR (nuclear hormone receptors), a class of molecules that plays a critical role in transcription [1]. These co-repressors mediate the down-regulation of gene expression and play important roles in the life and death choices that regulate normal development.

The nuclear hormone receptor (NHR) superfamily is a large family of mainly ligand-dependent transcription factors that play a role in the regulation of reproduction, growth, differentiation, and homeostasis. Members of the family share several structural features including a conserved DNA binding domain (DBD) that targets the receptor to hormone response element (HRE) sequences. The carboxyl-end of the receptors contains a ligand-binding domain (LBD) in which is embedded a hormone-dependent transcriptional activation domain. The LBD serves as a molecular switch that recruits co-activator or co-repressor proteins that regulate transcription of the target genes. Ligand-dependent receptors like the thyroid hormone receptor (T3R) and retinoic acid receptor (RAR) stimulate transcription when ligand is bound and repress it when the ligand is absent [2].

N-CoR (nuclear receptor co-repressor)[3] and SMRT (silencing mediator of retinoid and thyroid hormone receptors [4] were the first identified co-repressors. They fill overlapping but non-redundant roles in regulating transcription. Both SMRT and N-CoR exist in multi- protein complexes that have an estimated size of 1.5–2 mDa. A SMRT complex, isolated by a combination of conventional and immunoaffinity chromatography has been shown to contain histone deacetylase 3 (HDAC3) and transducin (beta)-like I (TBL1). The HDAC3-containing, SMRT and N-CoR complexes can bind to unliganded thyroid hormone receptors (T3Rs) *in vitro*[5]. Although both co-repressors are expressed widely, extensive hematological abnormalities, including blocks in erythrocyte and T-cell development [6], follow targeted deletion of N-CoR.

Co-repressors mediate transcriptional silencing by inhibiting the basal transcription machinery and by recruiting chromatin-modifying enzymes [2,5,7-11] Histone deacetylation, which produces a more compact chromatin structure that is inaccessible to transcriptional activators [8], appears to be the predominant means of chromatin modification. Studies of RAR and T3R show that ligand binding leads to the displacement of an HDAC-containing complex from the nuclear receptor in exchange for a histone acetyltransferase (HAT)-containing complex and this may serve as a general mechanism for switching nuclear receptors from a transcriptionally repressive to a transcriptionally active state [12]. Changes in repression correlate with alterations in the level of N-CoR and/or SMRT. These levels are regulated by both the rate of synthesis of the co-repressors and, more dramatically, by their rate of degradation. Targeted proteolysis of transcriptional co-regulators has been established as a mechanism for cell-specific regulation of gene transcription [13]. Although the composition of the repressor complex is not fully understood, both TBLR1 and a protein called TBL1 that is highly homologous to TBLR1, are present and in some cells, the extent of transcriptional repression correlates with the amount of TBL1 present [9,14,15].

We originally isolated TBLR1 as a 201 bp fragment in a cDNA library prepared from a bone marrow preparation highly enriched for human hematopoietic stem cells [16]. This fragment, which had no homology with any sequence in the GenBank, hybridized with RNA from K562 (a human erythroleukemia cell line) and a placental library and cDNAs prepared from these tissues were used as a source of mRNAs for subsequent analysis. 5' RACE identified an open reading frame (ORF) encoding a putative 514 a.a. protein. In this paper, we report the structure of the TBLR1 gene and analyze its pattern of expression. These results indicate that alternative splicing of the mRNA results in the formation of at least two protein isoforms (termed α and β). Over expression of TBLR1 interferes with cell replication. Like TBL1, TBLR1 forms a complex with nuclear co-repressors and appears to play a role in stabilizing the active co-repressor complex.

## Results

### The TBLR1 family

Human TBLR1 exists as two isoforms that differ at their carboxyl end. The original isolate (now termed TBLR1α [GenBank: NM024665] was characterized using 5' extension of an mRNA fragment identified by differential amplification of subtracted cDNAs [16]. This sequence contained a complete open reading frame (ORF) and *in vitro *translation of an RNA prepared from the full-length cDNA, yielded a product of the predicted size (data not shown). Because the size of the assembled sequence (3.9 kB) was significantly smaller than the 7.6 kB message identified by Northern blotting (see below), a series of primers were constructed for use in 3' RACE in an attempt to identify RNA sequences that extended beyond the poly A site that characterized the 3' end of the original molecule. While none of these attempts was successful, primers selected from the middle of the ORF (bridging exons 13 and 14) revealed the existence of two messages that were identical at their 5' ends but differed at their 3' ends. The second of these mRNAs is termed TBLR1β [GenBank: AF268194]. Examination of the genomic sequence encoding TBLR1 revealed that these were produced by the failure of recognition of the splice donor site that normally demarcates the end of exon 14. In the variant, the following intron is not removed from the transcribed mRNA. It is translated to produce the alternative beta form. Thus the two isoforms are identical through exon 14 but the 15^th ^exon of the beta variant is encoded by a sequence that begins immediately after exon 14 without an intervening splice. while the exon 15 of the alpha form is encoded by a sequence located 6000 bp down stream. The deduced amino acid sequences of the divergent carboxyl-ends are included in the Materials and Methods section.

The nucleic acid sequence encoding TBLR1α is highly conserved. At the nucleic acid level the similarity in the open reading frame between the human and mouse genes is 89% but interestingly, in the 3' UTR, the first 500 bp have the same degree of similarity (88%) as the open reading frame. The next several 100 bp contain several smaller regions (40 – 70 bp) of almost complete identity. The sequence of the human mRNA recorded in GenBank [GenBank: NM024665] is 3950 bp; the homologous murine sequence [GenBank: AF268195] containing the open reading frame is 1800 bp but the 3' end of this sequence shares a 400 bp overlap with the 5' end of a 1750 bp cDNA sequence [GenBank: BCO18512], suggesting that the full-length message is actually at least 3150 bp. The human [GenBank: NP078941] and mouse proteins are remarkably similar (98% identity).

**Figure 1:**
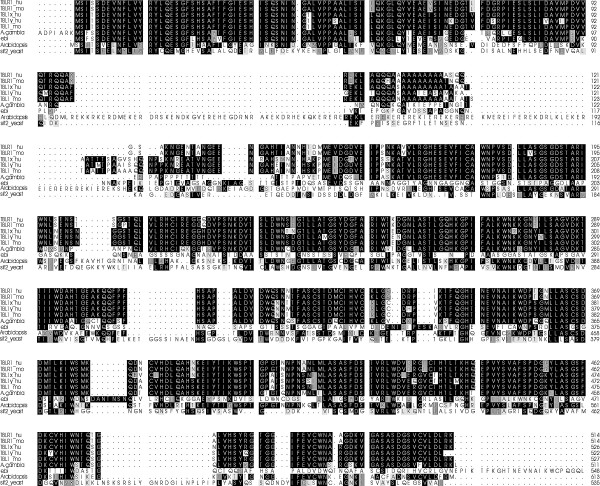
**Comparison of the amino acid sequence of TBLR1 with other family members and with the homologous proteins in other species**. The proteins shown in this alignment are: TBLR1-human.............................. NP078941, TBLR1-mouse............................... NP109657, TBL1X-human.............................. NP005638, TBL1Y-human............................... NP150600, TBL1-mouse.................................. XP135950, Ebi CG4063-PA [D. melanogaster] NP477329, A. gambia str. PEST....................... EA.A.12470, Arabidopsis; protein id: At5g........ NP201533, SIR4-interacting factor; SIF2p...... NP009661, Only proteins with homology to both the LisH domain (near the amino terminus) and the WD40 domains are included in the comparison. Regions of amino acid identity are shown in black; conservative substitutions are shown in gray. The large regions of non-homology are the result of insertions in the Arabidopsis and yeast sequences that have no counterparts in the other species.

TBLR1 has a high degree of similarity (90% identity or conservative substitution) to a pair of proteins encoded on the X and Y chromosomes (TBLX1 [GenBank: NP005638] and TBLY1 [GenBank: NP150600]. The mouse TBL1 protein [GenBank: XP135950] is equally well conserved (92% similar). These proteins are also highly homologous to a drosophila protein known as *Ebi *[GenBank: EA.A.12470; 85% similar] as well as to *Anopheles gambiae *[GenBank: EA.A.12470; 82% similar] and *Arabidopsis thaliana *[GenBank: NP201533; 73% similar] proteins. The yeast protein SIF2p, a SIR4 interacting factor [GenBank: NP009661] is 43% similar, indicating wide evolutionary conservation. Highly homologous proteins have also been found in chimpanzees, dogs, cows fish and chickens. The relationships among the proteins are illustrated in Figure 1.

The TBLR1 protein contains a series of WD40 repeats that are permuted with respect to the structural repeats (blades) of the β propeller domain. The amino-terminal end contains a perfect copy of the Lissencephaly type-1-like homology motif (LisH), an α-helical motif present in numerous WD40 repeat-containing proteins.

### Genomic structure of hTBLR1

**Figure 2:**
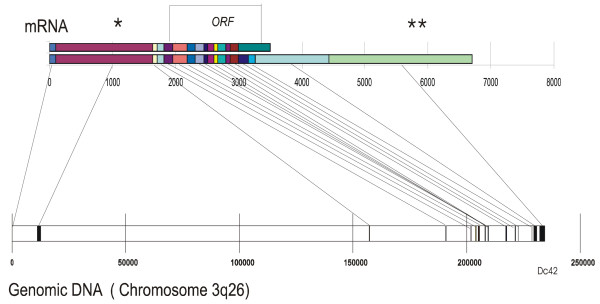
**Map of exons from TBLR1α and TBLR1β transcripts on the human chromosome 3q23 genomic sequence**. The sequences in the exon marked with asterisks were not included in the original description of TBLR1 and are not included in NM024665.2. The first sequence [GenBank: AK022268] (*) maps on chromosome 3 between the first 2 exons of TBLR1. The second sequence [**] is DC42 [AF267864] and maps immediately 3' to TBLR1. The actual 3'end of the gene has not been experimentally defined. mRNA numbering begins with the first nucleotide identified at the 5' end of the sequence and the genomic sequence is numbered from the corresponding base in the DNA. The sequence begins with bp 12127476 of [GenBank: NT_005962.15].

*TBLR1 *is located on chromosome 3 at 3q26. The gene occupies ~200,000 bp and the coding structure is assembled from 18 exons. Neither the first nor the last of these are included in the current Genebank assembly but are included here as result of Northern blotting evidence. The translational start site is located in the 5^th ^exon and is ~150 kb downstream from the first exon. The actual 3' end of the gene extends at least 2.2 kB beyond the sequence that we originally reported [16]. A probe synthesized to detect the hypothetical protein DC42 [GenBank: NM030921] which is located 3' of the previously characterized end of *TBLR1*, hybridizes with the mRNA encoding TBLR1 (see below). The sequence reported for *DC42 *encodes a putative small (102 a.a.) intronless protein that has no significant similarity to any other protein in GenBank from any organism. The mouse sequence is assembled from 16 exons and is located on chromosome 3 (between 3A2 and 3A3). The structure of the human gene is shown in Figure 2.

### Expression of TBLR1

**Figure 3:**
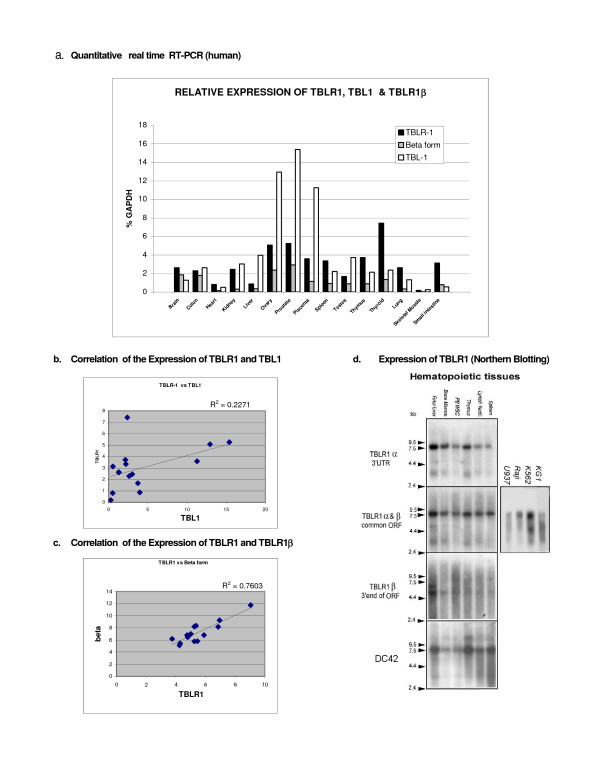
**Expression of TBLR1**. **a. Quantitative real time RT-PCR analysis of the expression of TBLR1, TBLR1β and TBL1 m RNA**. The data shown are relative concentrations of mRNA normalized to the concentration of GAPDH mRNA found in each tissue. **b. Correlation of TBLR1 & TBL1 Expression**. The data shown were extracted from Figure 3a and compare the level of expression of TBLR1 and TBL1. **c. Correlation of TBLR1 &TBLR1β Expression**. The data shown were extracted from Figure 3a and compare the level of expression of total TBL1 and TBLR1β. **d. Northern analysis**. Blots of human total RNA (Multiple Tissue Northern Blot™) or blots prepared using RNA extracted from several human hematopoietic cell lines were analyzed with ^32^P labeled probes as shown in the figure. The same filter was used for all the tissue hybridizations. Between hybridizations the bound radioactive probes were stripped and the filter exposed to fresh film to assure that the stripping had been complete. The autoradiographs were scanned to produce the images shown.

The expression of the messages for TBLR1, as well as that for TBL1X were examined using quantitative real time PCR. The results are shown in Figure 3a. Expression varies widely from tissue to tissue. In many tissues, including thymus, spleen, thyroid, lung and brain TBLR1 expression exceeded TBL1. On the other hand, in ovary, prostate and placenta, which have the highest overall levels of these mRNAs TBL1 greatly exceeded TBLR1. Little correlation was found between TBLR1 expression and TBL1 expression (Figure 3b) but expression of TBLR1β, the splice variant, generally correlated with the expression of the predominant alpha form (Figure 3c). The proportion of the splice variant varied from tissue to tissue but in no case was it the predominant form. Northern analyses with probes from different regions of the TBLR1 message (Figure 3d) produced complex results. A probe from the 3' untranslated region detects a 7.6 kb transcript in many tissues. Hybridization with a probe containing the 5' end of the ORF identified 3 mRNAs. In addition to the 7.6 kb band there is an abundant 3.9 kb message and a variably expressed band of ~4.9 kb that is most often detected in hematopoietic tissues. Mouse tissues show a similar pattern. An oligonucleotide probe specific for the 3' end of TBLR1β detected only the 4.9 kb mRNA. Hybridization with a probe designed to detect the theoretical protein DC42, which maps 2.2 kb downstream from the presumed 3' terminus of the message, hybridizes with a 7.6 kb message that appears to be identical to the one detected by probes that hybridize with the ORF. Hybridization with a probe designed to detect the cDNA sequence [GenBank: AK02 2268] also detects the 7.6 kb transcript of TBLR1. This sequence was identified in a human mammary cDNA library and maps in the large first intron deduced from the genomic sequence. It is not present in either our original isolate or any of the subsequently isolated clones. Its presence in the predominant transcript detected by Northern analysis of TBLR1 indicates the presence of additional splice variants of this gene. Hybridization with this probe also detects an ~3.7 kb RNA whose origin is unknown. No sequences that are homologous to [GenBank: AK022268] have been detected on the X or Y chromosomes where TBL1 is located.

**Figure 4:**
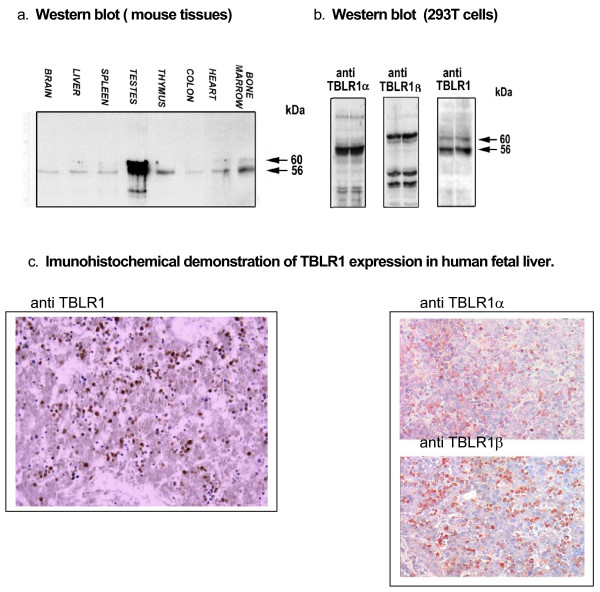
**a. Expression of TBLR1 in mouse tissues (Western Blotting)**. Anti peptide antibodies were made against deduced sequences from TBLR1. The antibody used was prepared against a sequence unique to TBL1 does not react with TBL1. After transfer, the membranes were stained with polyclonal rabbit anti-TBLR1 peptide antibodies and the transferred proteins visualized with HRP-labeled donkey anti-rabbit IgG. The bound HRP was detected using luminol as the substrate. **b. Expression of TBLR1 by HK293T cells (Western Blotting)**. TBLR1 proteins were isolated by immuno-affinity chromatography from lysates of HK293T cells. Rabbit anti-TBLR1(117-125), covalently coupled to the gel, was used as the immunoadsorbant. The bound material was eluted with a glycine-HCl buffer (pH 2.8) and then run on the gels. After transfer to nitrocellulose, these were stained with either anti TLR1α or TRLR1β or with anti anti-TBLR1(117-125) which detects both isoforms. The proteins were visualized as above. **c. Immunohistochemical Detection of the expression of TBLR1 in human fetal liver**. All of the samples were from 17 week fetuses. The sample on the left was stained with the anti-TBLR1 antiserum that detects both isoforms but does not detect TBL1. The staining was detected with HRP coupled goat anti rabbit IgG using DAB as the chromagen. The original magnification was 40×. A digital image was captured and cropped in Photoshop. The images on the right show samples stained with the specific anti α and β sera. They were also detected with HRP coupled goat anti rabbit IgG but using AEC as a chromagen. The images were captured on film and then scanned to produce the digital images shown. The original magnification was 40×. The hematopoietic cells of the fetal liver stained more intensely with the anti TBLR1β antiserum. All of the samples were counterstained with haematoxylin.

Western blots were prepared using three polyclonal anti TBLR1 peptide antibodies; antibodies directed against the carboxyl end of TBLR1α and TBLR1β and an antiserum specific for the region of maximal difference between TBLR1 and TBL1. This later antiserum does not react with TBL1 at all and enabled us to distinguish between TBLR1 and TBL1 in mouse tissues. TBLR1 was first isolated by immunoaffinity chromatography using the antiserum that distinguishes between TBLR1 and TBL1 and the eluted material was electrophoresed and then blotted with anti TBLR1α and anti TBLR1β antisera. The pattern produced by anti TBLR1α using mouse tissues is shown in Figure 4a. The anti TBLR1β antiserum is unfortunately not useful for western blotting. Despite our inability to detect the mouse beta form by western blotting, complex patterns that varied from tissue to tissue were observed when the antibody that detects both isoforms was used. A component with the molecular mass (~56 kD) predicted by the sequence of the ORF (Figure 4a) is present in most tissues but in some tissues, an additional component at ~60 kDa was also detected. The larger component was found in both mouse and human hematopoietic tissues (data not shown) and is prominent in extracts of mouse testes. The addition of soluble peptide to the diluted antiserum inhibited all of the staining (data not shown). In mouse bone marrow the predominant form is also a 56 kDa peptide. The highest levels of TBLR1 are present in fetal liver and immunohistochemical staining shows strong staining of hematopoietic cells in the developing liver (Figure 4c). The immunohistochemical results indicate that the predominant isotype in the fetal liver is the beta form.

The human kidney cell line 293T expresses readily detectable quantities of TBLR1 (Figure 4b). Anti TBLR1α produces a single band at ~56 kDa, while anti TBLR1β detects only a larger band at ~60 kDa. The results obtained with the antibody that distinguishes between TBLR1 and TBL1 are also shown in Figure 4b. This antibody, made with a peptide unique to TBLR1, is directed at an epitope shared by both the α and β isoforms and detects both bands. The results are particularly informative since they demonstrate; 1) more than one form of TBLR1 exists. 2) the β-isoform identified by 5'RACE and quantified by quantitative real time PCR, encodes an expressed protein; 3) a single cell type produces more than one isoform; and 4) the higher molecular weight form detected by the antibody to the C-terminal fragment of the β form is likely to be TBLR1 and not TBL1. Although the peptide used for the immunization of the animals that made the affinity absorbed antibody is not present in TBL1, the possibility that TBL1 forms a heterodimer with TBLR1 and is thus co-precipitated by anti TBLR1 has not been completely eliminated.

### Intracellular localization of TBLR1

**Figure 5:**
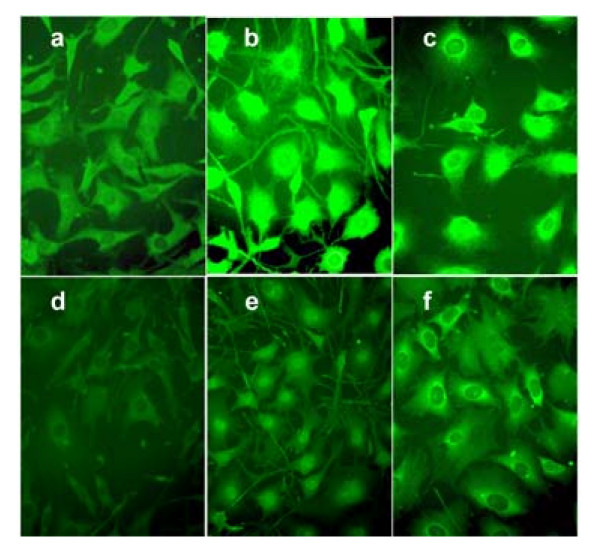
**Intracellular localization of TBLR1**. 3T3 cells respond to apoptotic stimuli with increased expression of TBLR1 and with translocation of TBLR1 into the nucleus. grown in on collagen coated cover slips were used. Untreated cells in the log phase of growth are shown in panels a and d. Apoptosis was induced by either serum starvation (incubation with DMEM containing 0.2% FBS for 16 h) [panels b and e] or growth with100 ng/ml of the topoisomerase inhibitor, camptothecin (CPT) [panels d and f]. The top panels [a, b, and c] were stained for total TBLR1 with anti TBLR1 [117-125] while the bottom panels [d, e, and f] were stained with anti TBLR1β. The images were obtained using a Leitz Orthlux microscope at an original magnification of 600 × with a Nikon 60X phase fluorescence objective (NA 1.4) and captured with a Nikon digital camera.

To determine the intracellular localization of TBLR1, the anti-peptide antibodies described above were used in an indirect immunofluorescence assay. The target cells were mouse 3T3 fibroblasts. The results are shown in Figure 5. In 3T3 growing under normal conditions, the staining is primarily cytoplasmic (Figure 5a and 5d). In serum-starved cells, the staining increased dramatically and nuclear (Figure 5b and 5e) and nuclear staining became prominent. After treatment with camptothecin (CPT; Figure 5d and 5f), the intensity of both cytoplasmic (with perinuclear intensification) and nuclear staining increased. Staining for total TBLR1 (panel a, b, and c) or just TBLR1β gave qualitatively similar results but anti TBLR1β staining was almost totally nuclear after serum starvation but remained predominantly cytoplasmic after CPT. The increase in nuclear staining suggests that under conditions of stress TBLR1 levels increase, and suggests a physiologic role for TBLR1 in the response to these stimuli.

### Interaction of TBLR1 with SMRT

**Figure 6:**
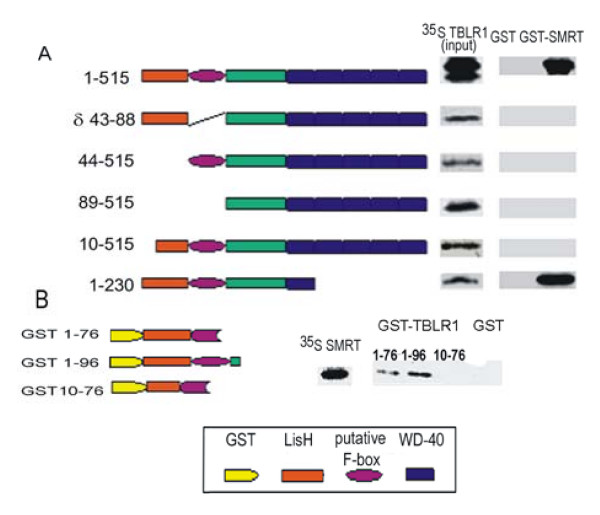
**Sequences required for the interaction of TBLR1 with SMRT**. a. TBLR1 constructs were cloned in-frame into pCDNA3 with an N-terminal Flag tag. b. HuSMRT cDNA (1-900) was PCR-amplified and cloned in-frame into PGEX-6p-1. EcoR1 linearized plasmids were purified and used as a template for *in vitro *translation using ^35^S methionine. Expression of the GST fusion protein was induced with IPTG and the product adsorbed onto Glutathione sepharose 4B. For the pull-down experiment, 5 ng of ^35^S labeled and 20 ul of GST agarose were mixed. After incubation the bound material was eluted and electrophoresed through a 10% SDS PAGE gel. 10% of the labeled material was reserved and electrophoresed separately to demonstrate the efficiency of the original labeling.

The interaction of TBLR1 with components of the transcription regulatory complex can be demonstrated by both GST "pull-down" and co-precipitation. Labeled TBLR1 prepared by in vitro translation using ^35^S methionine can be "pulled-down" by an immobilized amino terminal fragment of SMRT. As shown in Figure 6A, full-length TBLR1 is pulled down by GST SMRT(1-300) but does not bind to a control GST column. Truncated forms of TBLR1 lacking the F-Box (TBLR1 [δ 43-88]), the N-terminal 44aa (TBLR1 [89-515] or even just lacking the amino terminal 9aa (TBLR1 [10-515] are not pulled down by the truncated SMRT (Figure 6B.) indicating that both the putative F-box and the N-terminus are critical for a high affinity interaction of TBLR1 with SMRT. A fragment consisting only of the first 230 a.a.(TBLR1 [1-89] = TBLR1 [δC] i.e. lacking all of the WD40 repeats and the carboxyl terminus) was as efficient as the full-length protein in reacting with SMRT.

We have also confirmed that TBLR1 associates with HDAC3. In cells transfected with either his-tagged HDAC3 alone or his-tagged HDAC3 along with a flag-tagged TBLR1, the flag-tagged TBLR1 binds only to Ni-agarose columns that have bound HDAC (Data not shown).

### Effect of co-transfection of TBLR1 on SMRT and NCoR expression

**Figure 7:**
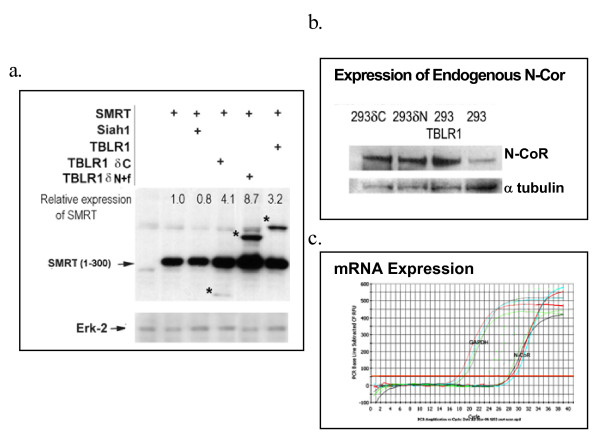
**Expression of TBLR1 alters the expression of SMRT and N-CoR**. a. **Transient expression of TBLR1 alters the expression of co-transfected SMRT(1-300)**. COS7 cells were co-transfected with pCDNA His-SMRT(1-300) and the His-tagged TBLR1 constructs described above. Control cells were transfected with pCDNA His-SMRT(1-300) and an empty vector.36 hrs after transfection the cells were lysed and equal amount of proteins were electrophoresed in SDS-PAGE gel. After transfer anti-His mAb was used to detect the transiently expressed proteins. The TBLR1 constructs used in this experiment were also His-tagged and are visible on the blot after staining. They are marked with * in the figure. The immuno-precipitates were quantified by phosphorimaging. After stripping, the blots were restained with anti ERK as a control for loading differences. b. **Stable expression of TBLR1 alters the expression of endogenous N-CoR**. Jurkat were infected with retroviruses expressing TBLR1 or the TBLR1 [δN] and TBLR1 [δC] construct. The retroviral vector also expressed GFP which was used to identify the infected cells. After two rounds of sorting the cells were analyzed by Western blotting for their expression of N-CoR. Th blot was then stripped and restained with an antibody to α tubulin as a loading control. c. **Stable expression of TBLR1 does not alter the expression of endogenous N-CoR or SMRT message**. mRNA was prepared from the cell lines used in Figure 3b and examined using quantitative real-time RT-PCR. Primer pairs that identified GAPDH and N-CoR were used. The results shown are representative of 3 experiments performed in triplicate. The samples are labeled as follows: black = control Jurkat; red = Jurkat (TBLR1); blue = Jurkat (TBLR1 [δC]); green = Jurkat(TBLR1 [δN])

Expression of the SMRT truncate is greatly increased in cells expressing both SMRT(1-300) and TBLR1. Truncated forms of TBLR1 (TBLR1 [δC] and TBLR1 [δ N+F-box] increase the level of SMRT(1-300) more than the wild type molecule (Figure 7a). The effect is seen with the fragment that binds SMRT (TBLR1 [δC]) and one that lacks the N-terminal binding site for N-Cor or SMRT (TBLR1 [δ N+F-box]). The expression of the TBLR1 mutants varied considerably after transfection. The greatest increase in SMRT(1-300) expression (8 fold) was produced by co-transfection with TBLR1 [δN], which was also the most highly expressed TBLR1 protein. However the next most effective protein was TBLR1 [δC] (4 fold increase in SMRT), which was the least expressed of the three TBLR1 proteins. Co-transfection with full-length TBLR1 resulted in an ~3 fold increase in SMRT(1-300). Co-transfection with siah-1, a factor known to contribute to the targeting of N-CoR for proteolytic destruction did not affect SMRT(1-300) expression. These results were obtained analyzing the transient expression of a SMRT fragment.

To confirm their physiologic relevance of these results, the experiment was repeated with cells engineered to stably express the TBLR1 constructs as a consequence of retroviral infection. The expression of endogenous N-CoR in these lines was examined using western blotting (Figure 6b). The results were similar to those found after co-expression of the TBLR1 truncates and the SMRT fragment. All of the lines that ectopically expressed TBLR1 or its truncates showed increased steady state expression of N-CoR To assure that the increase in protein expression was not a consequence increased mRNA expression, the expression of N-CoR message was assessed by quantitative real-time RT-PCR (Figure 7b). GAPDH was used as a control. The levels of N-Cor mRNA in cell lines that expressed TBLR1, TBLR1 [δC] or TBLR1 [δN] were indistinguishable from the control line.

### Effect of proteasome inhibitors on SMRT(1-300) expression

**Figure 8:**
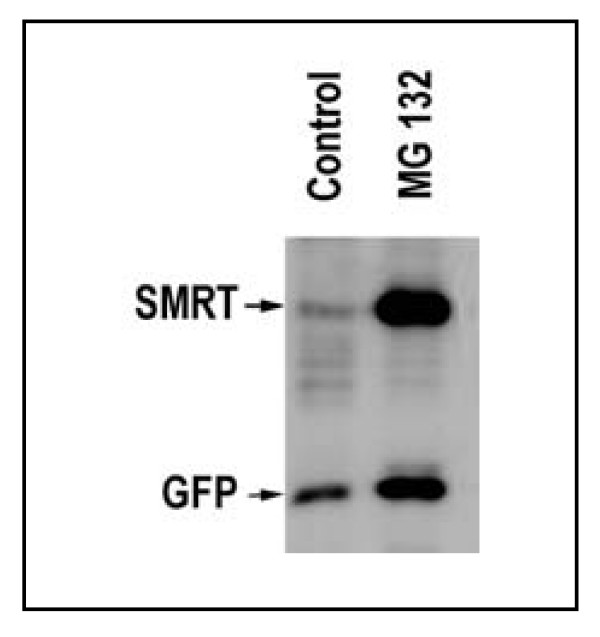
**Effect of proteasome inhibitors on SMRT (1-300) expression**. COS7 cells, expressing His -SMRT(1-300) and GFP-His_6 _were treated with 15 um MG132 for 10 hours. The His-tagged proteins were detected by Western blotting.

To demonstrate that the levels of SMRT(1-300) found in COS 7 cells are controlled by ubiquitination we treated the transfected cells with MG132 in DMSO or with DMSO as a control and measured the level of SMRT by Western blotting as described above. MG 132 (N-CBZ-Leu-Leu-Leu-al) is a potent, membrane-permeable proteasome inhibitor[17]. To control for differences in cell survival etc., the COS cells were co-transfected with a vector that expressed GFP along with the His-SMRT vector. GFP is not degraded via the ubiquitin-proteasome pathway. As shown in Figure 8, MG132 treated COS7 cells express levels of SMRT that are ~25 times those found in the DMSO control. Only a relatively small difference in GFP expression was observed.

### Effect of ectopic expression of TBLR1 on cell growth

**Figure 9:**
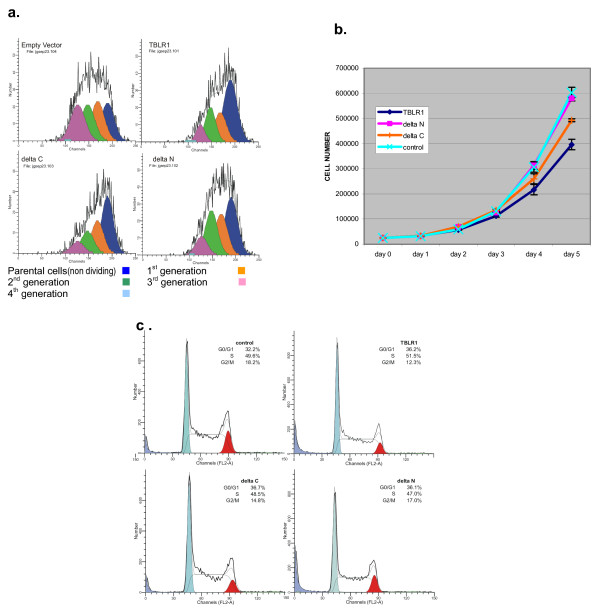
**Growth inhibition after transfection with TBLR1**. **a. DiI staining of HK293T cells 72 hours after transfection with TBLR1 constructs**. The cells were stained with DiI prior to transfection. The lipophilic dye distributes to both daughter cells after division and the loss of the dye reflects the number of times the cells have divided. The color filled curves are the computer generated model of the components of the mixed population. **b. Growth of JURKAT cells over-expressing TBLR1**. JURKAT cells infected with retroviral constructs expressing only GFP or GFP and a TBLR1 construct were seeded at a concentration of 25,000 per ml in 12 well Costar plates. Each day the entire contents of a well was removed and counted using a Coulter Model Zf cell counter. Each culture was performed in triplicate and the mean and S.D have been plotted. **c. Cell cycle parameters (DNA content) of log phase JURKAT cells ectopically expressing TBLR1**. Cells were harvested while in log phase of growth (72 hrs after plating at 50,000 cell per ml) and stained with PI as described. Fluorescence from PI bound to DNA was analyzed using a BD FACScan cytometer and the list mode data analyzed using ModFit (Verity Software) to calculate cell cycle parameters.

Transient expression of TBLR1or the truncated form lacking the WD40 domains in HK293 T cells leads to growth arrest. After transfection almost half of the cells that expressed full-length TBLR1 or the truncated form lacking the C-terminal half of the molecule (TBLR1 [δC]) fail to undergo a single round of mitosis within 3 days. This non-proliferative fraction, which was 0.24 after the control transfection, increased to 0.48 (TBLR1); 0.46 (TBLR1 [δC]) and 0.36 (TBLR1 [δN]) after transfection with TBLR1 constructs. The proportion of cells that divided 3 times (4^th ^generation) was reduced from 25% after transfection with the control vector to between 9 and 11% after transfection with TBLR1 or its truncates. The interference with growth was apparent even among cells that escaped the initial block. The proliferative index (a measure of the growth of the cells that escaped the initial growth arrest) decreased from 2.18 after transfection with the empty vector to 1.54 (TBLR1); 1.56 (TBLR1 [δC]) and 1.75 (TBLR1 [δN]). The results are illustrated in Figure 9a and summarized in Table 1. Transfection with TBLR1 [δN] produced a more modest block than transfection with either the fill-length material or TBLR1 [δC].

**Table 1:** Proliferation HK293T after transfection with TBLR1

	Empty Vector	TBLR1	Delta C	Delta N
Parent	23.80%	48.40%	45.30%	36.20%
Generation 2	25.50%	20.40%	25.40%	25.40%
Generation 3	24.40%	21.10%	17.70%	27.00%
Generation 4	25.20%	8.90%	9.60%	10.80%
Generation 5	0.70%	1.10%	1.50%	0.50%
Generation 6	0.40%	0.00%	0.20%	0.00%
				
Proliferation Index	2.18	1.54	1.56	1.75
Fraction of non-dividing cells	0.24	0.48	0.46	0.36

Stable ectopic expression of TBLR1 also alters the growth properties of Jurkat cells. The growth rate of cells infected with retroviruses containing the same constructs used in the transient experiments is shown in Figure 9b. In 5 independent experiments, cells over expressing full-length TBLR1 or the truncated form lacking the C-terminal half of the molecule (TBLR1 [δC]) show a significant reduction in growth rate. Cells expressing TBLR1 [δN] grew at a rate that was statistically indistinguishable from cells infected with a virus containing only GFP. To determine the basis for the reduced growth rate, cells in the log phase of growth, were stained with propidium iodide to determine their DNA content. The results (Figure 9c) show a consistent reduction in the proportion of cells in the G2/M phase of the cell cycle in cells expressing either full length TBLR1 or TBLR1 [δC]. This decrease in G2/M cells is associated with increases in the proportion of cells in both the G0/G1 and S phases of the cycle, suggesting that over-expression of TBLR1 can contribute to both G0/G1 and S phase arrest. This results is consistent with the known effects of HDAC (to which TBLR1 binds) and chromatin remodeling complexes (which contain TBLR1) in regulating cell division. [18-21].

## Discussions and conclusion

TBLR1 gene is expressed widely and Northern analysis, using sequences from the ORF as a probe, revealed size heterogeneity of the expressed mRNAs (3.9, 4.7 kb and ~7.3 kb). These are due to both the presence of alternatively spliced messages and the presence of multiple polyadenylation sites. The 4.7 kb form is more abundant in hematopoietic tissues. In addition to the heterogeneity in message size, the protein also has several isoforms. The isoforms are produced by the use of alternative splice donor sites. TBLR1β, differs from the original sequence at the carboxyl-terminus (3' end of the ORF). The mouse homologue shows mRNA size heterogeneity that is similar to the human RNA. Both the human and mouse genes have a complex and somewhat unusual genomic structure. The human gene contains 18 highly conserved exons spread over 230 kb of genomic DNA. The first and second exons are ~140 kb upstream of the third, which in turn, is separated from the fourth exon by 33 kb of intronic DNA. The translational start site is located an additional 11 kb downstream in the middle of the fifth exon. The gene also has an extremely long 3'UTR that includes a sequence deposited in GenBank as DC42. This is described as an intronless gene for which there is no independent evidence of expression and may not actually encode a functional message. Human TBLR1 maps to chromosome 3q26 while mouse TBLR1 is located between 3A2 and 3A3.

The broad evolutionary conservation of this gene family is of note. Homologs are present in yeast, plants, fish and flies as well as in mammals and the insect proteins are > 80% similar to the mammalian ones. No homology was detected to prokaryotic genomes.

The cDNAs for TBLR1 encode members of the β-transducin or WD-repeat family [22]. Eight WD-repeats are present in both the human and mouse proteins. The splice site that distinguishes between TBLR1α and TBLR1β is immediately 3' of the last WD-repeat sequence signature. At the nucleotide level, the sequence of TBLR1α has 79% homology to Homo sapiens mRNA for transducin (β)-1 like protein (TBL1) [GenBank: Y12781]. The only homology of TBLR1 and TBL1 to the prototypic signal transducing guanine nucleotide binding regulatory (G) protein β sub- unit is in the WD-repeat domains. TBL1 has been mapped to the X chromosome and deletions in the region containing TBL1 are associated with adult onset sensorineural deafness[23]. Both TBLR1 and TBL1 are homologous to a Drosophila protein called *ebi *The regions of maximal nucleotide conservation between TBL1 and *ebi *are at the N-terminal end of the molecule and in the WD40 repeats. The C-terminal ends of these proteins are not homologous. The carboxyl terminal exon of TBLR1α has some homology to several members of the Arp2/3 complex of proteins that control actin polymerization [24]. The equivalent region of TBLR1β has no homology to any known protein. The N-terminal 100 amino acids of TBLR1 co-precipitates with both HDAC3 and SMRT. The data indicate that TBLR1 binds directly to SMRT, which in turn binds HDAC3. SMRT binding of HDAC3 plays an important role in regulating chromatin structure and gene expression[5,25,26] The homologous region of TBL1 also co-precipitates with these co-repressors [9,10] and has been reported to form an effective transcriptional repressor. This portion of the molecule contains a LisH motif. LisH is a β-propeller-binding motif and is thought to mediate the dimerization of WD-40 proteins [27-29] Sif2p, the yeast homolog of TBLR1, is a tetramer and contains an unusual eight-bladed β-propeller structure, that apparently mediates tetramerization [30]. Mutations in conserved LisH amino acids significantly reduced the half-life of TBL1 and altered its intracellular localization. TBL1 mutated in the LisH domain was not imported into the nucleus [31]. Our data indicates that elimination of the LisH domain prevents interaction with SMRT.

The actual role that the TBLR1 family plays in cell physiology remains unclear. Like the proverbial blind man trying to describe an elephant, each laboratory has found a role for family members in their own system. Presumably, TBL1 and TBLR1 are multi-functional proteins. We found TBLR1 in a screen for messages over-expressed in early hematopoietic cells [16]. TBL1 deletions are associated with deafness [23]. The drosophila homolog, *ebi *has been reported to regulate epidermal growth factor receptor signaling [32] promote the degradation of a repressor of neuronal differentiation (Ttk88), and to limit S phase entry. A cDNA, described as the "human homologue of *ebi*" was reported to encode a protein that plays a role in the ubiquitin pathway [33]. More recently, both TBL1 and TBLR1 were reported to be required for transcriptional activators of nuclear hormone receptors and other regulated transcription factors [34]. Cofactor exchange through ubiquitin-dependent protein degradation was proposed to account for the transcriptional activation dependent on TBL1 and/or TBLR1. Yoon *et al. *[35] were unable to find any evidence for transcriptional activation in their studies, which postulated a role for TBL1/TBLR1 in histone code reading. Knock-down experiments using small interference RNA (siRNA) techniques indicate that TBL1 and TBLR1 are functionally redundant but essential for repression by unliganded thyroid hormone receptors [9]. Tomita *et al. *used the frog oocyte system to demonstrate that unliganded TBLR1 interacts with T3R and recruits TBLR1 to its chromatinized target promoter *in vivo*, accompanied by histone deacetylation and gene repression and showed that the recruitment of TBLR1 or related proteins is important for repression by unliganded T3R [11]. Yoon *et al. *have demonstrated that there is an additional site of interaction between a site near the most N-terminal of the WD-40 repeats of TBL1 and the RD4 region of N-Cor [9]. These interaction sites on SMRT flank the SANT motifs and it has been suggested that they play a role in interpreting the histone code and promoting histone deacetylation [36]. Expression of a TBLR1 fragment that contains this interaction site, but lacks the C-terminal two-thirds of the molecule including the WD-40 repeats, leads to a partial proliferation arrest, suggesting again, the multiplicity of roles that these family member play [18-21].

The proteins of the TBLR1 family also contain variant F-box near the N terminus of the molecule. F-box proteins, in combination with *Skp*1 and Cullin, form part of an E3 ubiquitin ligase that plays a critical role in the targeting of phosphorylated proteins for ubiquitination and subsequent proteasomal degradation. Because proteolysis is irreversible, proteasomal degradation provides a unidirectional regulatory switch. The initiation of DNA replication, chromosome segregation, and exit from mitosis are all triggered by the destruction of key regulatory proteins [37] [38-39] The F-boxes in TBLR1, TBL1 and *ebi *all lack a tryptophan near the NH2-end of the motif that is associated with *Skp1 *binding to F-box proteins that recognize phosphorylated protein [40]. A cDNA, described only as the "human homologue of *ebi*" encodes a protein that the authors believe plays a role in the ubiquitin mediated destruction of β-catenin [33] and they suggest that this *ebi *can bind *Skp*1 and is a key component in a pathway that targets unphosphorylated proteins for ubiquitination and subsequent proteasomal degradation [33] [41].

Ectopic expression of full-length TBL1 potentiates repression by unliganded T3R. TBL1 does not bind directly to T3R; it binds to SMRT and/or N-CoR and this interaction was reported to contribute an autonomous repression function to the complex. The methods used in these experiments could not distinguish among the possibilities; 1) that TBL1 changed the structure of N-CoR, perhaps by altering the accessibility of the SANT motifs, producing increased repression, 2) that it increased the quantity of N-CoR present in the target cells or 3) that it facilitated the recruitment of an additional co-repressor. We have shown that over expression of TBLR1 or either the C- or N-terminal truncates increases the expression of NHR co-repressors. Co-transfection of TBLR1 with SMRT(1-300) leads to greatly enhanced expression of a truncated SMRT in a transient expression model and cells in which TBLR1 is stably over expressed have elevated levels of endogenous N-Cor. Co-transfection with the wild type TBLR1 as well as both the N- and C-terminal truncates leads to a significant increase in both SMRT(1-300) and endogenous N-CoR expression. Thus, both ends of the molecule can independently contribute to co-repressor stabilization. Alternatively, the region between a.a. 80 and 175, and shard in all of our constructs) may play an autonomous role in increasing co-repressor expression. Since SMRT(1-300) is degraded via a ubiquitin-mediated mechanism and inhibition of ubiquitination leads to elevation of SMRT, our data suggest the possibility that TBLR1 may act by preventing the degradation of nuclear co-repressors. This stabilization appears to be part of a complex regulatory network that governs the level of unliganded nuclear receptor. Although this result is the opposite of what might have been predicted on the basis of the results described above for *ebi*-mediated targeting of β-catenin [33]both sets of data suggest that the TBLR1 family interacts with proteins subject to proteasomal degradation and the actual outcome, protection or targeting, may depend on either the nature of the target protein or the identity of the other proteins that interact with TBLR1. It may also be controlled by which of the TBLR1 family members does the interacting.

The mechanism through which the steady state level of SMRT and N-CoR expression is regulated is not obvious. The truncated construct of TBLR1 [TBLR1[δN] containing the C-terminal half of the molecule lacks the sequence involved in binding to the RD1 region of the co-repressors but has the greatest effect on both SMRT and N-CoR expression. Although this fragment contains most of the sequence that Yoon *et al. *suggested is responsible for binding the RD4 region of the co-repressor, the SMRT(1-300) fragment that we used lacks this RD4 region. Thus can not identify the binding partner responsible for the increased co-repressor levels found in cells over-expressing TBLR1. It may be that oligimerization of TBLR1 is required co-repressor stabilization and that a heteromeric multimer of a truncated form with the wild type may form a complex with SMRT that is an unsuitable substrate for proteasomal degradation. Pulse-chase experiments with co-transfected SMRT(1-300) suggest that ectopically expressed TBLR1 [δN] stabilizes SMRT (data not shown) but the pulse chase experiments do not provide an explanation for the increased level of SMRT expression associated with co-transfection of either the N-terminal fragment of TBLR1 or the whole molecule.

The fluorescence studies show that the intracellular distribution of TBLR1 is influenced by the metabolic state of the cells. The increase in nuclear staining may either represent translocation to the nucleus or nuclear trapping. In either case, it is likely that the TBLR1 that accumulates in the nucleus is degraded with different kinetics than the cytoplasmic material and that differential localization as well as alterations in proteasomal targeting, are the explanation for the effect of increased TBLR1 expression on co-repressor levels.

These results suggest that the TBL1-TBLR1 family plays a role in the regulation of N-Cor and/or SMRT expression; the amino terminal end docks the molecule to the co-repressor, perhaps altering its intracellular localization and targeting it for degradation. The carboxyl end appears to contribute to this activity but also appears to be the source of tissue specificity.

In summary, we have identified TBLR1 as a transcriptional regulator interacting with the co-repressors of nuclear hormone receptor activity. We have cloned the gene encoding this protein and identified it as a member of a small family of proteins that include at least two isoforms encoded by the same gene and closely related, X- and Y linked proteins, called TBL1X and TBL1Y. The evidence suggests that these proteins act by altering the stability of the co-repressor complex and that they may also contribute, or recruit other proteins that provide, an autonomous repressor function.

## Methods

### Cell culture, reagents and plasmids

COS7 cells are grown in Dulbecco's modified eagle medium (DMEM) with 10% fetal bovine serum (FBS) at 37°C, 5% CO2. MG132 was purchased from Sigma Chemical (St. Louis, MO.). PcDNA4.1His/Max and PGEX6p-1 were purchased from Invitrogen and Pharmacia respectively. TBLR1 cDNA and a variety of truncated derivatives were amplified from PcDNA.TBLR1 plasmid using proof reading Taq polymerase. A truncated SMRT cDNA SMRT(1-300) containing the first 900 nucleotides of the message and encoding the amino terminal 300 a.a. of the 2473 a.a. protein was amplified from a human placenta cDNA library. This fragment contains the RD1 domain of SMRT but lacks RD2 and RD3. It also lacks the C-terminal ID domains as well as both SANT domains [36]. PCR products were cloned in frame into PcDNA4.1His/Max for mammalian expression or *in vitro *translation and cloned in frame into PGEX6p-1 plasmid for bacterial expression.

### Northern analysis

Multiple Tissue Northern Blot™ membranes were purchased from Clontech. Hybridizations were performed with [α^32^P]dATP labeled probes that also contained a modified dCTP to facilitate removal of the hybridized probe so that the blot can be hybridized repeatedly (Strip-ez™, Ambion Inc, Austin TX 78744).

### Real Time Quantitative PCR (rtqPCR)

A panel of total RNA from human tissues (First Choice Human Total RNA) was purchased from Ambion, Inc. Real-time RT-PCR was performed using a SYBR Green PCR kit (SuperArray) and the iCycler Real-Time PCR Detection System (BioRad). PCR was conducted at 95°C for 5 min, followed by 40 cycles at 95°C for 60 seconds, 55°C for 30 seconds, and 72°C for 40 seconds. Data was collected at 72°C, and a melting curve analysis was done at the end of the cycling program to verify the quality of PCR products. In some experiments the PCR products were collected and electrophoresed through a 1.0% (W/V) agarose gel to confirm the product size. Except for those used to identify TBLR1β, the primers were purchased from Super Arrays Inc and had the characteristics listed in Table 2. The primer pair used to identify TBLR1β had the sequence: sense 3'-CTGCCTCACCATTTGGTTGT; 3'-ATCTGCAAAACCGTTGGAAA.

**Table 2:** 

Gene Symbol	RefSeq Accession #	Reference positions	Band Size (bp)	Gene Symbol
TBL-1	TBL1X	NM_005647.2	2185–2203	114
TBLR-1	IRA1	NM_024665.2	474–494	159
N-CoR	NCOR1	NM_006311.2	7531–7549	149
SMRT	NCOR2	NM_006312.1	6820–6840	83
GAPD	GAPD	NM_002046.2	162–183	108

### Preparation of cell lines ectopically expressing TBLR1

Jurkat cells were used to produce cells lines that stable expressed TBLR1 and its variants TBLR1, TBLR1 [δN] and TBLR1 [δC] constructs were made using proof reading PCR. Each sequence was amplified from templates with EcoR1 and Xho1 sites on the 5' and 3' ends. The amplified products were digested and the fragments were cloned into the MIG vector provided by Dr. David Baltimore. This vector contains an IRES site downstream of the cloning site and this is followed by a sequence encoding green fluorescent protein (GFP). Phoenix 293-Ampho cell line were used to produce the viruses. They were cultured in 10% FCS DMEM to 80% confluence and transfected with 3 μg of each construct and 1 μg of VSVg using Lipofectamine-2000 in the presence of 2 ug/ml of polybrene. The medium was changed at 24 hours. 72 hours after the transfection, the medium containing virus was collected and used to infect the Jurkat cells. Five days after infection, each Jurkat cell line was sorted for GFP positive cells. The sorted green cells were maintained in 10% FCS RPMI medium. The expression levels of the expressed proteins were confirmed by Western blotting.

### Anti TBLR1 antibodies

To facilitate analyzing the expression of the TBLR1 proteins, the deduced peptide sequences were used to prepare anti-peptide antibodies directed against the non-identical portions of the two isoforms. Rabbits were immunized with the carboxyl terminal peptides of TBLR1α and TBLR1β. The deduced sequences of the C-terminal peptides are:

TBLR1 α TQTGALVHSYRGTGGIFEV**CWNA(C)AGDKVGASASDGSVCVLDLR**

TBLR1 β TQV**CLHYLNGQVLLNLGRSI**CLYTLPHHLVVIPLVALIELLVLK

The rabbits were immunized with the peptides shown in bold face above coupled to ovalbumin in Freund's Adjuvant (CFA) and boosted with the same antigens in Incomplete Adjuvant every 2 weeks for a total of 4 injections. Antibody titers were measured by ELISA using both the immunizing peptide and the recombinant TBLR1. Since the peptide used to produce the antibody for TBLR1α is identical to the homologous peptide found in TBL1, we also prepared an antibody directed against the region of TBLR1 (amino acids 117–125; **AASQQGSAK**) that differed most from TBL1. The resultant antibody was then absorbed with a peptide (CGVSHQNPSK-amide coupled to acrylamide) representing the equivalent sequence in TBL1, to further reduce cross-reactivity. The resultant antibody was then affinity purified using the immunizing peptide. The site recognized by this antibody is 30 amino acids downstream of the putative F-box in the amino terminal half of TBLR1.

### Western blotting

1.0–2.0 mg of tissue or 10–20 million cells were lysed with SDS-lysis buffer and the lysates denatured at 100°C for 5 minutes. Samples were electrophoresed through either 10% SDS gels, transferred to nitrocellulose membranes, blocked with 5% non-fat milk and stained with the primary antibody at the dilution recommended by the distributor. The membrane was washed and stained with a secondary, horseradish peroxidase-labeled conjugate. After washing, the membranes were exposed to a detection cocktail containing hydrogen peroxide, phenol, and luminol and the light emitted by the HRP/hydrogen peroxide catalyzed oxidation of luminol, was detected by blue-light sensitive autoradiography film. In some experiments the TBLR1 proteins were isolated by immuno-affinity chromatography using the reagents provided by Pierce Chemical in their Seize Primary Immunoprecipitation kit. Rabbit anti-TBLR1(117-125) described above was covalently coupled to the gel. The bound material was eluted with a glycine-HCl buffer (pH 2.8) and then run on the gels.

### Immunofluorescence

Mouse 3T3 cells were grown in DMEM with 10% fetal bovine serum. washed, fixed with 3.8% paraformaldehyde, permeabilized with 0.0.5% Triton-PBS and blocked with 10% human serum in PBS. They were then incubated with either anti-TBLR1 [117-125] or anti TBLR1β antibody (1:25) and stained with FITC-F(ab')2 anti-rabbit IgG antibody that had previously been absorbed to render them unreactive with human immunoglobulins (Jackson ImmunoResearch Laboratories, Inc., West Grove, PA, USA 19390). Normal rabbit serum was used as a control.

### Cell cycle parameters

Cells in log phase growth were washed, resuspended in PBS and fixed in PBS paraformaldehyde (0.25%final). After 15 min at room temperature the cells were washed once with PBS and the pellet suspended in cold methano (-20°C)l. After 20 minuutes the cells were again washed aresuspended in PBS containing RNAse (100 ug/ml) After 20 minutes at 37°C the samples were resuspended in propidiumiodide (PI) at 10 ug/ml. The cells were analyzed after 90 minutes of staining.

### Immunohistochemistry

Slide-mounted tissue sections are deparaffinized and hydrated through graded alcohols to running water. All slides are treated with 3°/v hydrogen peroxide in methanol for 30 min to remove endogenous peroxidase activity. All 3 antibodies were used at a final concentration of 1:25 and detected with HRP (horseradish peroxidase) coupled goat anti rabbit IgG that had been absorbed to reduce reactivity with human immunoglobulins (Jackson ImmunoResearch Laboratories). Normal rabbit serum was used as a control.

### Co-immunoprecipitation

COS7 cells were transfected using lipofectamine (Gibco). After 48 hours, the cells were collected and proteins extracted with RIPA buffer. The protein extracts were incubated with the primary monoclonal antibody for 4 hours at 4°C and the resulting complexes bound to protein A/S agarose beads. After washing with RIPA buffer, the beads were resuspended in loading buffer and boiled for 5 minutes to elute the proteins. These proteins were used for Western blotting.

### Growth arrest after transfection

HK293T cells were stained with 50 uM of the lipophilic carbocyanine, DiI (Molecular Probe cat# V-22885), in serum free DMEM for 8 minutes at 37°. After 3 washing with warm medium 200,000 cells were transferred to each well of a 6 well cluster plate. The cells were transfected with either an empty PCDNA4 control vector or PCDNA4 derived vectors containing TBLR1, TBLR1 [δN] or TBLR1 [δN] using Lipofectamine 2000 (Invitrogen). 4 μg of plasmid was used for each transfection. After 48 and, 72 hours, the cells from each well were harvested and analyzed for the expression DiI. The data was analyzed using the "Proliferation Wizard" included in ModFit LT for Win32 (Verity Software House, Topsham, ME 04086).

### Sequence analysis

Plasmid DNA was sequenced by the chain termination method in the Core Sequencing Facility of NYU Cancer Institute using an Applied Biosystems automated sequencer. The resulting sequences as well as the ESTs that were identified using the NCBI BLAST program were assembled using Sequencher software (Gene Codes Corp., Ann Arbor, MI) under a license to the Research Computing Resources of the NYU Medical Center.

### GST pull-down experiment

^35^S labeled proteins were synthesized *in vitro *using a T7 *in vitro *translation kit from Promega. GST and GST fusion proteins were induced with 0.1 mM IPTG and purified on glutathione-agarose column. To perform pull-down experiments, 10 ul of ^35^S labeled proteins were mixed with 20 ul 50% agarose coupled with GST or GST fusion proteins in 150 ul PBS. The reaction was incubated with frequent rotation for 2 hrs at 4°C, and then washed extensively with PBS, containing 0.1% NP40. The beads were resuspended in SDS-PAGE loading buffer, boiled for 5 minutes to elute the bound proteins which were electrophoresed on 10% SDS-PAGE gel and visualized with a phosphorimager.

## Competing interests

The author(s) declare that they have no competing interests.

## Authors' contributions

X-MZ isolated and sequenced TBLR1. He performed the pull-down experiments described here. LZ performed all of the real time PCR analyses. JG carried out the Western blots.SB did the sequence alignment and the homology analysis. CQ performed the cell cycle analysis and contributed to the design of some the experiments. RSB conceived of the study, and participated in its design and coordination and helped to draft the manuscript. All authors read and approved the final manuscript.
